# *In Situ* Measurement of Nanoparticle–Blood Protein Adsorption and Its Heterogeneity with Single-Nanoparticle Resolution via Dual Fluorescence Quantification

**DOI:** 10.1021/acs.nanolett.4c01469

**Published:** 2024-07-22

**Authors:** Yuanyuan Niu, Yingjie Yu, Xinyang Shi, Fangqin Fu, Hai Yang, Qiang Mu, Daniel Crespy, Katharina Landfester, Shuai Jiang

**Affiliations:** † Key Laboratory of Marine Drugs, Chinese Ministry of Education, School of Medicine and Pharmacy, Ocean University of China, Qingdao 266003, China; ‡ Laboratory for Marine Drugs and Bioproducts, Qingdao Marine Science and Technology Center, Qingdao 266237, China; § Department of Pharmacy, Qingdao Central Hospital, University of Health and Rehabilitation Sciences, Qingdao 266042, China; ∥ The First Department of Breast Surgery, Qingdao Central Hospital, University of Health and Rehabilitation Sciences (Qingdao Central Medical Group), Qingdao 266042, China; ⊥ Department of Materials Science and Engineering, School of Molecular Science and Engineering, Vidyasirimedhi Institute of Science and Technology (VISTEC), Rayong 21210, Thailand; # Max Planck Institute for Polymer Research, Ackermannweg 10, 55128 Mainz, Germany

**Keywords:** protein corona, nanomedicine, drug delivery, nano−bio interaction, Vroman effect

## Abstract

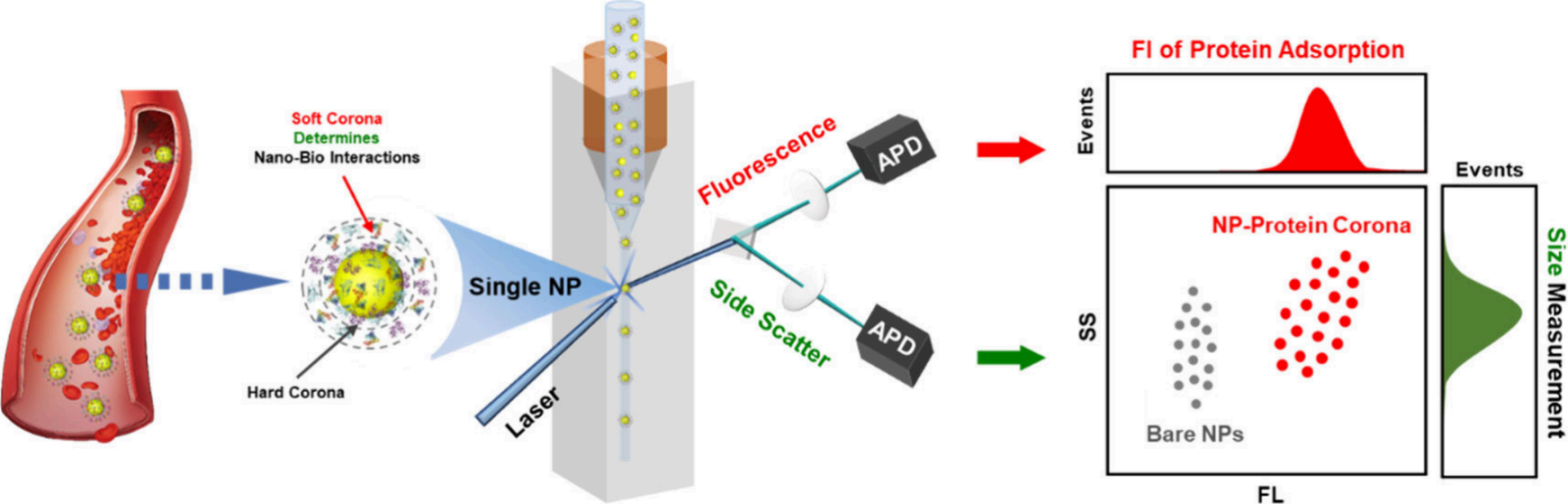

The formation of a protein corona gives nanomedicines a distinct biological identity, profoundly influencing their fate in the body. Nonspecific nanoparticle–protein interactions are typically highly heterogeneous, which can lead to unique biological behaviors and *in vivo* fates for individual nanoparticles that remain underexplored. To address this, we have established an *in situ* approach that allows quantitative examination of nanoparticle–protein adsorption at the individual nanoparticle level. This method integrates dual fluorescence quantification techniques, wherein the nanoparticles are first individually analyzed via nanoflow cytometry to detect fluorescent signals from adsorbed proteins. The obtained fluorescence intensity is then translated into protein quantities through calibration with microplate reader quantification. Consequently, this approach enables analysis of interparticle heterogeneity of nano–protein interactions, as well as *in situ* monitoring of protein adsorption kinetics and nanoparticle aggregation status in blood serum, preconditioning for a comprehensive understanding of nano–bio interactions, and predicting *in vivo* fate of nanomedicines.

Once nanoparticles (NPs) enter the bloodstream, proteins spontaneously adsorb onto their surface, forming a protein corona (PC) that imparts them a new biological identity.^1−3^ Unraveling NP–protein interactions is essential for predicting the *in vivo* fate of nanomedicines and promoting their clinical translation. Current PC analysis methods mainly rely on preisolating NP–PC complexes from surrounding medium, resulting in a loss of loosely bound outer-layer proteins (soft corona) that directly interact with biological interfaces.^4^ Despite considerable efforts devoted to *in situ* characterization, such as dynamic light scattering,^5,6^ fluorescence correlation spectroscopy,^7,8^ and DOSY ^19^F-NMR^9,10^ for investigating NP size evolution, circular dichroism spectroscopy^11,12^ for protein structure change, and isothermal titration calorimetry^13,14^ for protein adsorption thermodynamics, these methods only provide data based on the average of the nanoparticles population. The heterogeneity of NPs and their differential protein adsorption profiles, which lead to distinctive *in vivo* fates among individual NPs, remains underexplored.

Achieving single-particle analysis of NPs is a precondition for resolving the heterogeneity of the NP–protein interactions. Scanning and transmission electron microscopies,^15−18^ atomic force microscopy,^19^ super-resolution fluorescence microscopy,^20,21^ and multicolor stimulated emission depletion microscopy^22^ have been employed; however, high throughout analysis is a limit. Recent advancements include 3D real-time single-particle tracking spectroscopy, which can “lock-on” to individual freely diffusing polystyrene NPs to probe their protein coronas.^23^ By analyzing fluorescence signals and diffusive motions of tracked NPs, researchers quantified the “hard corona” using mean-squared displacement analysis. Another innovative approach is scattering microscopy-enabled real-time all-optical nanoparticle analysis, which tracks the formation of protein corona in full serum at single-particle level without labeling.^24^ This method allows real-time *in situ* tracking of adsorbed protein mass, affinity, and kinetics on metallic and dielectric nanoparticles. The heterogeneity of protein corona has been examined using magnetic levitation, which demonstrated that exposing NPs to human plasma results in a highly heterogeneous corona composition.^25^ This technique also enabled extraction of homogeneously coated NPs as well as monitoring of PC evolution on the NP surface in a few hours. Despite these encouraging advancements, achieving high-throughput and universal analysis of protein adsorption at single-nanoparticle level remains challenging but is crucial for distinguishing interparticle heterogeneity.

Flow cytometry is a high-throughput single-particle detection technique that allows quantitative analysis of cell-size particles in suspensions.^20,26^ Due to its sensitivity limitation, conventional flow cytometer can detect only large particles (>500 nm) with strong fluorescence (>200 fluorescent molecules).^27^ Flow cytometry has been applied to investigate NP–protein interactions through immunolabeling of adsorbed protein corona, allowing detection of surface molecular motifs (e.g., ApoB-100 and IgG epitopes) presented for biological recognition, such as cell receptors.^28^ However, due to limited detection sensitivity, a flow cytometer can only measure nanoparticle populations, providing no information at a single NP level. Consequently, the heterogeneity of protein interactions among the individual NPs cannot be distinguished.

In this study, we established a nanoflow cytometry (NanoFCM)-based approach that enables *in situ* quantitative probing of NP–protein interactions and the heterogeneity among individual NPs at single-nanoparticle level (Scheme 1). NanoFCM integrates light scattering with single-molecule fluorescence detection in a sheathed flow, allowing detection of single NPs as small as 24 nm for silica and 7 nm for gold nanoparticles at a throughput up to 10,000 particles per minute.^29,30^ Moreover, the fluorescence detection limit of NanoFCM is down to three fluorescent molecules of Alexa fluor 555 per particle. Using this technique, we monitored size evolution and aggregation state of amino-functionalized polystyrene NPs (PS-NH_2_ NPs) upon incubation in protein solutions. By fluorescently labeling the proteins, we directly quantified the adsorption of human serum albumin (HSA) and dual proteins (HSA and transferrin, Tf) on the NP surface and analyzed the heterogeneity of protein adsorption among individual NPs. Furthermore, protein adsorption kinetics in human serum was directly monitored without separation.

**Scheme 1:**
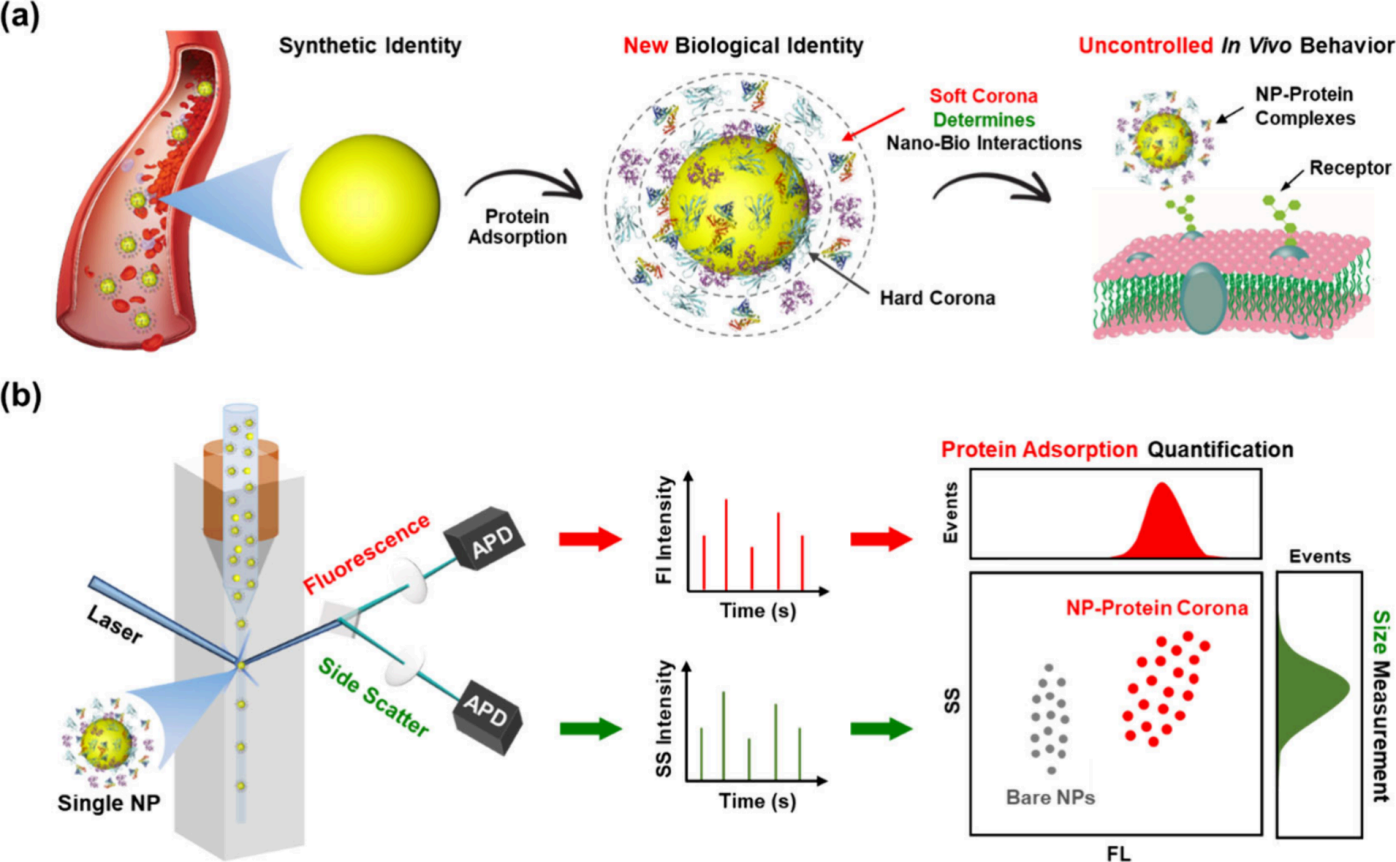
Schematic Illustration of the Formation and Biological Effect of Protein Corona on Nanoparticles (a) and the *in Situ* Characterization Technique for Detecting Nanoparticle–Blood Protein Interactions at Single-Nanoparticle Resolution Using NanoFCM (b)

The principles of NP–blood protein interactions and their *in situ* detection are schematically described in Scheme 1. Human serum or HSA solutions were incubated with NPs to mimic the protein environment in the blood. The resulting mixtures, comprising newly formed NP–PC complexes, were directly subjected to NanoFCM analysis, without isolating them from free (nonadsorbed) proteins and NPs.

NanoFCM, also known as single-molecule flow cytometry, enables the individual analysis of NPs by integrating light scattering with single-molecule fluorescence detection. NPs are linearly aligned to pass through a laser beam enabled by a high-velocity sheath flow, which can hydrodynamically focus the sample flow into a very fine stream (∼1.4 μm). As each NP traverses the central region of the focused laser beam, it generates a burst of photocurrent on both the side scattering (SS) and fluorescence (FL) detection channels. The burst area, representing the integrated number of detected photons, is recorded for each event.^31^ To the best of our knowledge, NanoFCM has to date not been used to investigate protein interactions with NPs. Regarding the NP–PC system, variation in SS signals indicates changes in NP size due to protein adsorption, while FL signals demonstrate the binding of fluorescently labeled proteins to individual NPs. Quantitative determination of the size of NP–PC complexes and number of proteins absorbed on individual NPs was achievable through corresponding calibration curves, established by using a series of monodisperse standard NPs with various diameters to calibrate SS intensity of NPs and employing fluorescence analysis to calibrate FL intensity of adsorbed proteins (Scheme S1).

PS-NH_2_ NPs were prepared by miniemulsion polymerization.^32^ Dynamic light scattering (DLS) revealed a hydrodynamic diameter (*D*_h_) of ∼150 nm with a low PDI of 0.037 for the NPs (Figure 1a). SEM and TEM micrographs showed NPs with uniform and spherical structures (Figure 1b,c). The NPs were then subjected to NanoFCM analysis, and the FL and SS signals were collected. Signals detected in the SS channel (Figure 1d) suggest a median particle size of ∼135 nm, calculated using the calibration curve shown in Figure S3. No signals were observed in the FL channel (Figure 1e) because this NP, serving as a control, was neither fluorescently labeled nor adsorbed with proteins. As shown in Figure 1f, the bivariate dot-plot of SS signals versus FL signals demonstrates the capability of multiparameter detection for individual NPs. This result aligned well with DLS, SEM, and TEM results, highlighting the exceptional sensitivity of NanoFCM for NP size detection.

**Figure 1:**
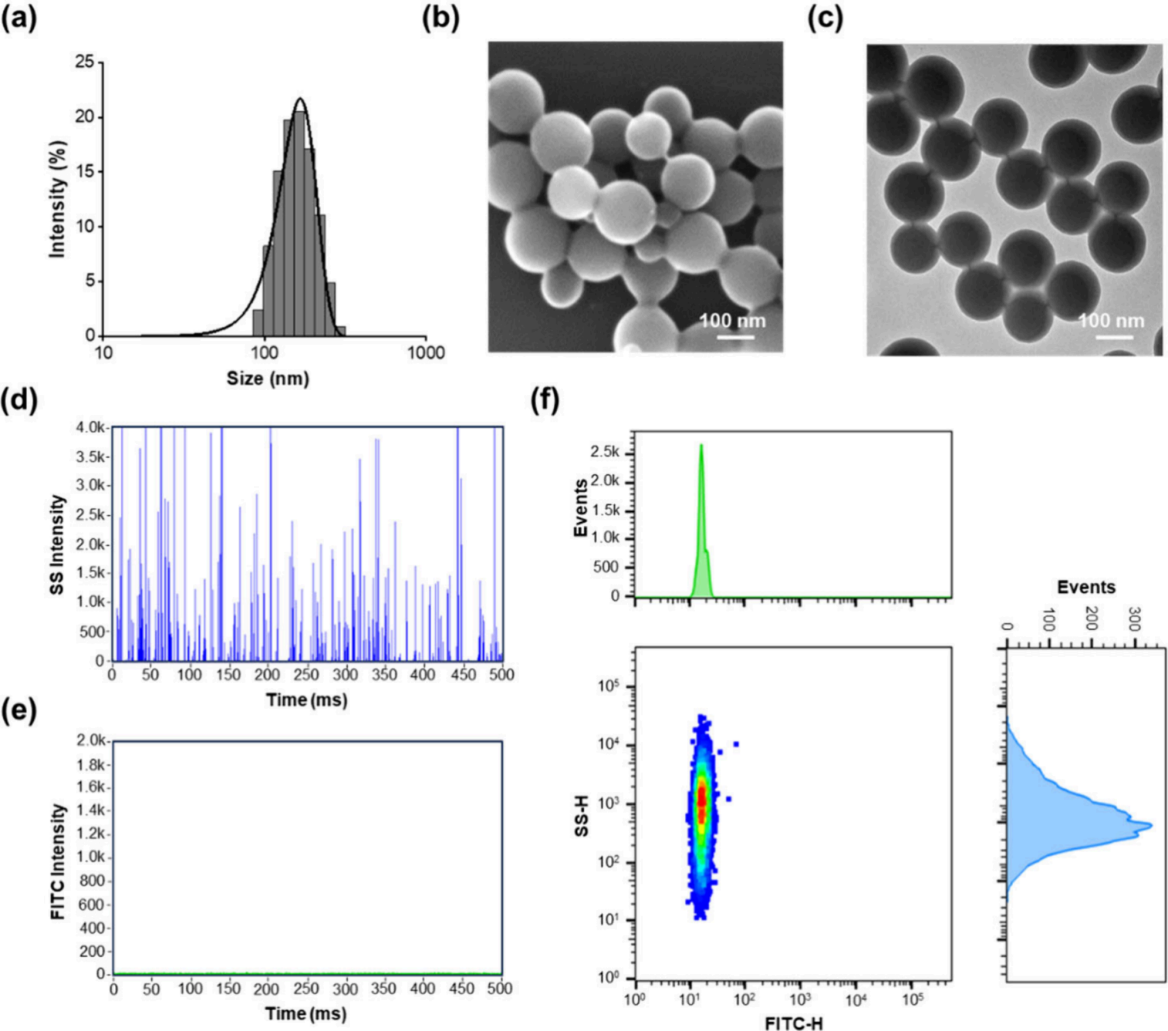
Characterization of the polystyrene NPs prepared by miniemulsion polymerization. (a) Size distribution histogram of NPs measured by using DLS. (b) SEM and (c) TEM micrographs of the NPs. Representative burst traces of concurrent detection of (d) SS signals and (e) FL signals of NPs. (f) Histograms and bivariate dot-plot of SS burst height versus FL burst height for polystyrene NPs.

Figure S4a demonstrates the successful covalent binding of FITC to HSA molecules, with excess unreacted FITC effectively removed. The structure of HSA was not affected due to the presence of 5–10 vol% DMSO in the labeling solution, as verified by the identical CD spectra before and after fluorescent labeling (Figure S4b). NPs were then incubated with HSA-FITC for 1 h to achieve an adsorption equilibrium. The resulting mixtures, containing newly formed NP–PC complexes, were directly analyzed by using NanoFCM without separation. The HSA molecule has a molecular weight of 66 kDa and an equilateral triangular prism shape with the dimensions of ca. 8 nm × 8 nm × 3 nm (Figure 2a).^33−35^ As the most abundant serum protein, HSA is frequently detected as a major component of protein corona.^23^

**Figure 2:**
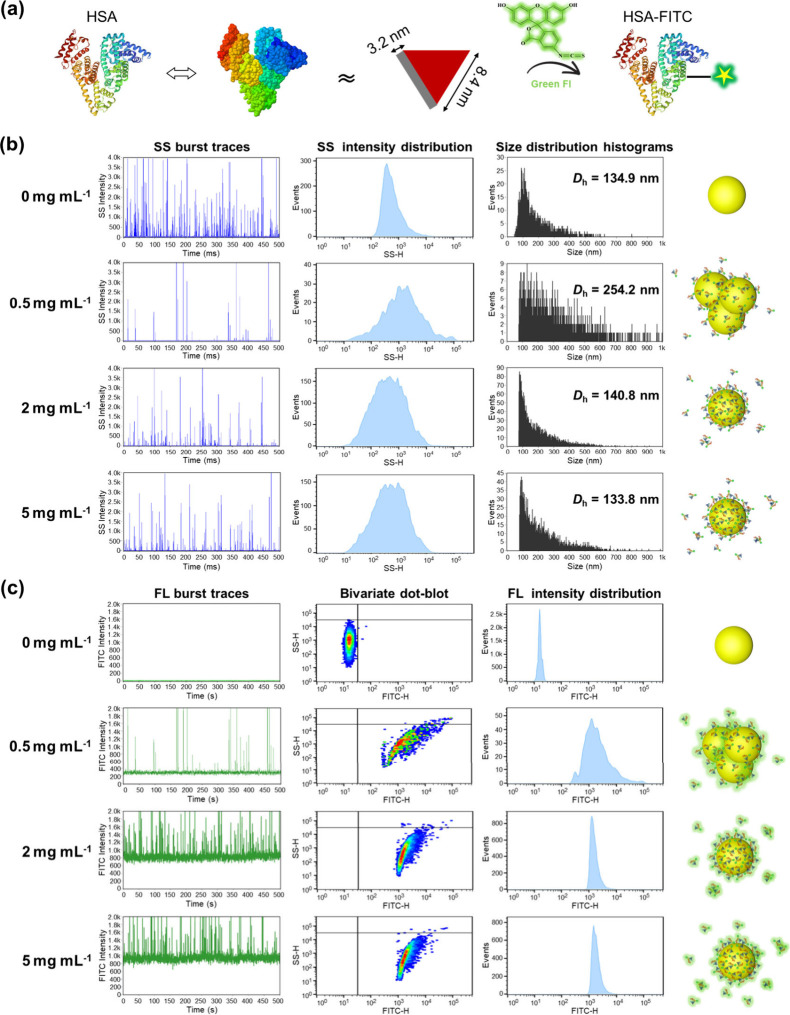
*In situ* detection of NP–protein adsorption by using NanoFCM. (a) Scheme illustrating the structure, 3D dimensions, and fluorescent labeling of HSA (Protein Data Bank (PDB) accession code 1AO6). (b) SS analysis of NPs before and after incubation with varying concentrations of HSA-FITC (0 mg mL^–1^, 0.5 mg mL^–1^, 2 mg mL^–1^, and 5 mg mL^–1^) for 1 h, including SS burst traces, SS intensity distribution histograms, and size distribution histograms. Size distribution was derived from the standard curve depicted in Figure S3a. (c) FL analysis of NPs before and after incubation with varying concentrations of HSA-FITC (0 mg mL^–1^, 0.5 mg mL^–1^, 2 mg mL^–1^, and 5 mg mL^–1^) for 1 h, including FL burst traces, bivariate dot-plots, and FL intensity distribution histograms.

NPs were incubated with HSA-FITC solutions at concentrations of 0 mg mL^–1^, 0.5 mg mL^–1^, 2 mg mL^–1^, and 5 mg mL^–1^, giving rise to protein corona formation around NPs. Sodium dodecyl sulfate-polyacrylamide gel electrophoresis (SDS-PAGE) (Figure S5) and TEM (Figure S6) both confirmed the presence of adsorbed HSA on the NPs. The samples containing NP–PC complexes together with free proteins were directly subjected to NanoFCM analysis, while carrying out DLS measurements as a comparison.

Figure 2b presents the SS burst traces, SS intensity distribution, and size distribution of NPs subjected to varying concentrations of HSA. The SS burst traces revealed detectable side-scattered light signals. The SS intensity and size distribution of NP–PC complexes exhibited initial broadening, followed by narrowing with increasing HSA concentration. At an HSA concentration of 0.5 mg mL^–1^, median particle size of NP–PC complexes reached ∼254 nm, doubling that of pure NPs (∼ 135 nm) (Figure 2b). The observed NP aggregation at this low HSA concentration was attributed to neutralization of positive charges of NPs with HSA,^36^ which will be explored in subsequent sections. Upon further increasing HSA concentrations to 2 mg mL^–1^ and 5 mg mL^–1^, the median particle size of NP–PC complexes was reverted to be identical with that of the pristine NPs (Figure 2b). This phenomenon results from a gradual increase of protein adsorption, causing an increasing surface negative charge on the NPs (isoelectric point of HSA ∼ 4.7).^37^ Consequently, electrostatic repulsion dominated and rendered the NPs colloidally stable. Comparable findings were corroborated by DLS studies, as depicted in Figure S7. These results underscore the efficacy of NanoFCM for *in situ* detection, enabling the prediction of *in vivo* NP aggregation stemming from blood protein adsorption.

Besides light scattering, fluorescence signals of NPs can be detected simultaneously (Figure 2c). FL burst traces exhibited negligible fluorescence signals in the case of bare NPs. Due to the small size of HSA, no detectable signals originating from free albumin were observed in NanoFCM analysis. As the concentration of HSA-FITC increased, a consistent rise in fluorescence signals of NPs was evident, indicating adsorption of HSA on NPs. Likewise, bivariate dot-plot diagrams and FL intensity distribution histograms demonstrated a clear rightward shift in NP population compared to those in bare NPs. At an HSA-FITC concentration of 0.5 mg mL^–1^, a population of larger NPs appeared in the upper right quadrant of the dot-plot, implying NP aggregation due to protein adsorption. In contrast, the fraction of these larger NPs was diminished as protein concentration increased to 2 mg mL^–1^ and 5 mg mL^–1^, suggesting that increased protein adsorption enhanced colloidal stability of NPs. In the literature, adsorption of serum albumins was found to be effective to stabilize NPs prior to biological experiments.^38^ The FL intensity showed no further increase from incubation with 2 mg mL^–1^ to 5 mg mL^–1^ of HSA, indicating the saturation of protein adsorption (Figure 2c).

The FL distribution exhibited a Gaussian pattern upon incubation with 0.5 mg mL^–1^ HSA. However, only a fraction of the signals was detected at HSA concentrations of 2 mg mL^–1^ and 5 mg mL^–1^, resulting in the loss of NPs with a lower fluorescence intensity (Figure 2c). This discrepancy can be attributed to the rising baseline of background fluorescence signal with increasing protein concentration, obscuring the weak signals of NPs. In particular, FL signals with an intensity exceeding 300 were detected at an HSA concentration of 0.5 mg mL^–1^, whereas only signals surpassing 900 and 1000 were detected at protein concentrations of 2 mg mL^–1^ and 5 mg mL^–1^, respectively. The presence of abundant free fluorescent proteins gave rise to an increase of background signals, leading to a loss of signals from NPs with low fluorescence intensities. Therefore, diverse strategies were subsequently implemented to mitigate the influence of free proteins, including (i) reducing FITC labeling ratio, (ii) purifying NP–protein mixture through dialysis, and (iii) purifying NP–protein mixture via centrifugation. In comparison to the fluorescence baseline of the direct detection method (depicted as a dashed line in Figure S8a), all three methods led to a reduction in the baseline to various degrees. Lowering the FITC labeling ratio only resulted in a slight decrease of baseline intensity, while both the dialysis and centrifugation dramatically lowered the baseline. Notably, centrifugation yielded minimal background signals.

Influence of baseline reduction on the detection capability for distinct NP populations was assessed through bivariate dot plots (Figure S8b). First, a quadrant gate was set at a FITC intensity value of 1k, designating signals with intensities below 1k as weak signals (found in the fourth quadrant, Q4) (Figure S8b). In comparison to the signals obtained via direct detection (Figure S8b1), a similar outcome was achieved by reducing the fluorescent labeling ratio (Figure S8b2). In comparison, dialysis led to a 9.4% increase in the detection rate of weak signals (Figure S8b3). Remarkably, centrifugation for purifying free proteins facilitated the detection of 37.5% weak signals (Figure S8b4), attributed to the nearly zero baseline (Figure S8a4). Consequently, the mild centrifugation method was employed in subsequent experiments.

The NPs were incubated with various concentrations of HSA-FITC, reaching a maximum of 50 mg mL^–1^ to mimic human blood content, followed by centrifugation to eliminate unbound proteins.^39^ Compared with pristine NPs, fluorescence signals exhibited a rightward shift with increasing protein concentrations within the 0–50 mg mL^–1^ range (Figure 3a–e). Notably, at 0.5 mg mL^–1^ of HSA incubation, the fluorescence intensity values exceeded those obtained at 2 mg mL^–1^ (Figure 3b,c), indicating that NP aggregation was probably caused by a reduction in electrostatic interactions due to neutralization of the positive charge on NPs with HSA. No significant change occurred when the HSA concentration was further increased to 5 mg mL^–1^ (Figure 3d), implying saturation of protein adsorption at 2 mg mL^–1^ of HSA incubation. However, at 50 mg mL^–1^ HSA concentration, fluorescent NP species with higher SS and FL intensities appeared (Figure 3e), which suggests possible aggregation of NPs in concentrated protein medium due to the bridging effect of protein molecules. Consistently, the SS intensity of NP–PC complexes displayed a pronounced rightward shift at concentrations of 0.5 and 50 mg mL^–1^, as opposed to that at intermediate protein concentrations. This shift was accompanied by a broadening of the size distribution, as depicted in Figure S9, validating the occurrence of NP aggregation at these specific concentrations. We used coefficient of variation (CV) to quantify the heterogeneity of size and protein adsorption amount of individual NPs. CV quantifies the dispersion of the data relative to the mean. Small CV values indicate that the data are tightly clustered around the mean, while large CV values indicate that the data are more spread out.^40^ CV of NP fluorescence upon incubation with varying concentrations of HSA-FITC revealed that the heterogeneity of NP–protein complexes increased as aggregation occurred (Figure 3i). The NP–protein complexes are relatively uniform in the nonaggregation state.

**Figure 3:**
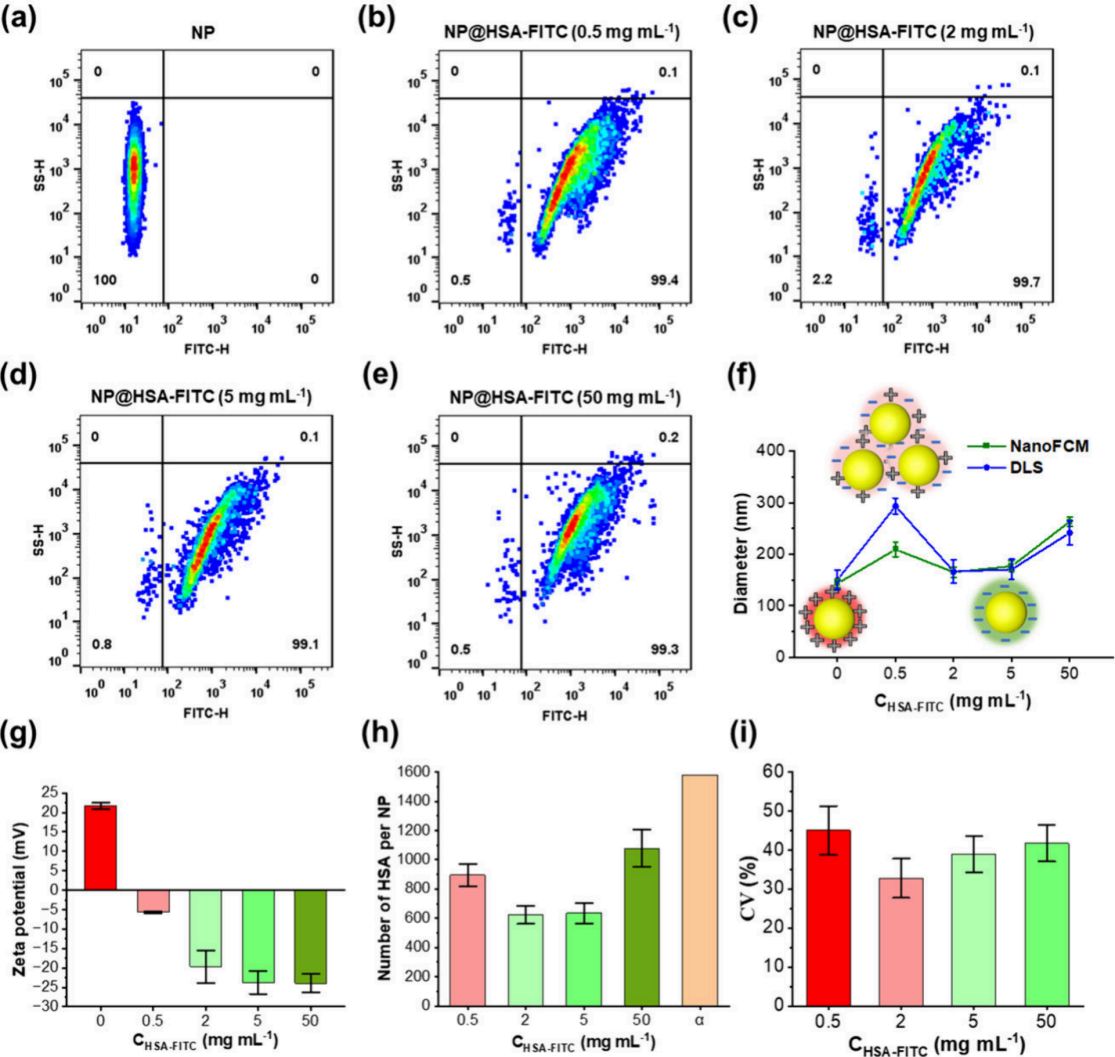
Characterization of protein adsorption on NPs at single-nanoparticle level. (a–e) Bivariate dot-plots of SS signals versus FL signals of NP–protein complexes upon incubation with varying concentrations of HSA-FITC (0 mg mL^–1^, 0.5 mg mL^–1^, 2 mg mL^–1^, 5 mg mL^–1^, and 50 mg mL^–1^). (f) Diameter of NP–protein complexes measured using DLS and NanoFCM. (g) ζ-potential of NP–protein complexes measured using a Malvern Zetasizer. (h) Calculated number of HSA adsorbed per NP, where α represents the theoretical monolayer saturation adsorption by considering the HSA molecules as a rigid sphere of diameter (*d*) = 7.5 nm and cross-sectional area (σ) = π*r*^2^ = 44.18 nm^2^. (i) Coefficient of variation (CV) of the fluorescence of NPs upon incubation with varying concentrations of HSA-FITC.

The results obtained with NanoFCM were validated through DLS studies, as depicted in Figure 3f. The ζ-potential of NPs underwent a transition from positive to negative after HSA incubation, due to the negative charge from adsorbed proteins (Figure 3g). At 0.5 mg mL^–1^ of HSA incubation, protein adsorption was relatively low, resulting in a nearly neutral surface. In this condition, the average particle size detected by DLS was approximately 300 nm, showing an increase of about 100 nm compared to that of pristine NPs. This phenomenon is likely due to the dominance of van der Waals short-range attraction over Coulombic electrostatic repulsion, resulting in slight NP aggregation. As the HSA concentration increased, protein adsorption gradually intensified, leading to elevated surface negativity of NPs. As the electrostatic repulsion took precedence, the NPs became more stable, and their sizes gradually approached that of pristine NPs (Figure 3f,g).

Analyzing at the single-particle level requires precise quantification of the HSA content on each NP surface. In theory, considering NPs as rigid spheres allows one to calculate their surface area. The maximum theoretical number of adsorbed proteins on the surface, denoted as σ, can be estimated to be around 1600 HSA per NP when HSA is perfectly packed as a monolayer. As shown in Figure 3h, the actual number of adsorbed proteins on the NP surface is consistently below the theoretical number α after incubation at all HSA concentrations, suggesting that HSA is sparsely distributed on the NP surface as a monolayer. This conclusion was further supported by the comparable protein amounts of hard corona (HC: 594 HSA per NP) and full corona (FC: 623 HSA per NP) (S10), indicating tight protein adsorption on NPs with minimal loss during the repetitive centrifugation steps to prepare the HC. The results obtained from NanoFCM were validated through the BCA assay, as depicted in Figure S2. BCA results showed that the number of adsorbed proteins initially increased and then reached saturation as the protein concentration increased. The obtained protein adsorption quantities were identical with NanoFCM results. The observed differences at low (0.5 mg/mL) and high (50 mg/mL) protein concentrations are due to the consideration of NP aggregation in the NanoFCM analysis but not in the BCA test.

To evaluate the applicability of our method for detecting protein adsorption in more complex systems, a solution of human serum albumin (HSA) and transferrin (Tf) in a 1:1 molar ratio was prepared. HSA, the most abundant plasma protein (∼55%),^41^ and Tf, a key iron transport protein,^42^ are major components found in protein corona of nanoparticles.^8^ NanoFCM analysis was used to study the adsorption of fluorescently labeled HSA (HSA-FITC) and Tf (Tf-Cy5) on PS NPs, as shown in Figure 4. The bivariate dot-plots of SS signals versus FL signals (Figure 4b,c) showed fluorescence signals for both HSA and Tf, indicating their adsorption on PS NPs. The double-positive signals in Figure 4d confirm the simultaneous presence of both proteins on the same NPs. Calibration of the FI results from NanoFCM with a microplate reader (Figure S1) revealed that approximately 409 Tf and 214 HSA molecules were adsorbed per NP (Figure 4e). The differing CV values for HSA and Tf suggest distinct adsorption behaviors. The higher adsorption heterogeneity of Tf compared to HSA is probably due to their different binding affinities and sizes (Figure 4f).

**Figure 4:**
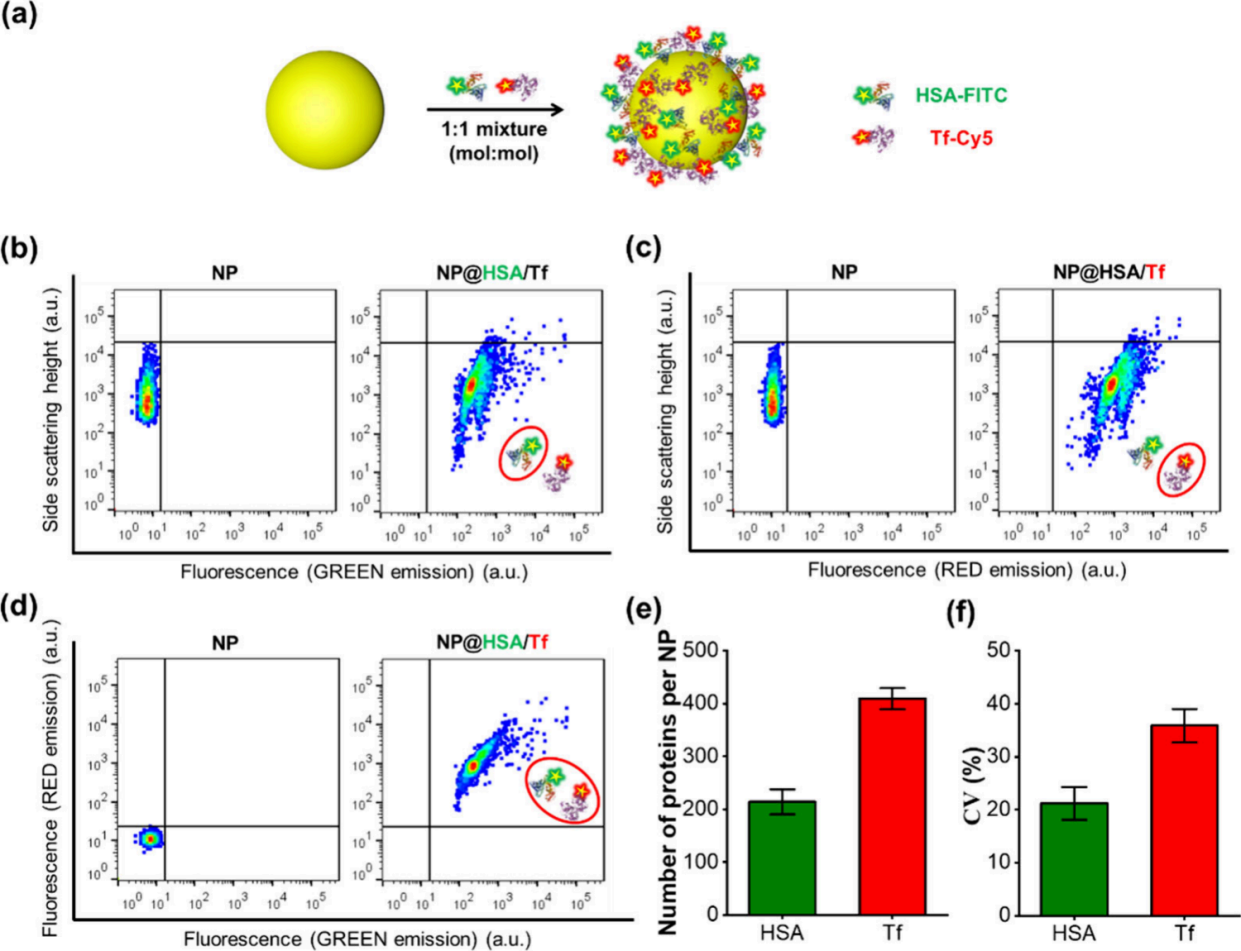
Protein adsorption analysis in a dual-protein system using NanoFCM. (a) Schematic illustration of protein adsorption in a model dual-protein system (HSA-FITC and Tf-Cy5). (b) Bivariate dot-plots of SS signals versus green FL signals representing HSA in PS@HSA/Tf. (c) Bivariate dot-plots of SS signals versus red FL signals representing Tf-Cy5 in PS@HSA/Tf. (d) Bivariate dot-plots of red FL signals versus green FL signals of PS@HSA/Tf. (e) Quantitative analysis of average numbers of HSA and Tf adsorbed per NP. (f) Respective CV values reflecting the adsorption heterogeneity of HSA and Tf in the complex system.

To prove the applicability of this technique for detecting smaller nanoparticles, silica nanoparticles (SiO_2_ NPs) with a diameter of ∼50 nm determined by TEM were synthesized and measured using NanoFCM. The results showed a size distribution ranging from 50 to 100 nm and a median particle size of ∼60 nm. After incubation with HSA, the NP diameter increased to ∼70 nm (Figure S11a,b). From the bivariate dot-plots of SS signals versus FL signals, we can clearly see a fluorescence increase on individual NPs due to protein adsorption (Figure S11c–e).

The Vroman effect, a pivotal phenomenon in protein corona research, describes the dynamic adsorption of proteins on NP surfaces, showcasing the competitive displacement of initially adsorbed proteins by others with higher affinities over time. Understanding this effect is crucial for unraveling the complexities of protein corona formation and its implications, as it governs the evolving composition and biological interactions of the protein corona, thereby influencing the fate of NPs in biological systems. However, achieving real-time analysis of the composition of the protein corona remains a critical challenge. Here, we contribute to *in situ* and quantitative monitoring of protein adsorption kinetics. While this does not elucidate specific protein exchange behaviors, it aids in understanding the Vroman effect.

The NPs were incubated with human serum (HS), and the protein adsorption kinetics were studied using NanoFCM. In Figure 5a, the schematic cross-section illustrates three representative phases of protein corona formation: adsorption and association phase, hard corona (HC) formation stage, and soft corona (SC) formation stage. Figure 5b demonstrates a time-dependent rightward shift in fluorescence signals of NPs upon incubation with the serum, a trend corroborated by the corresponding FL burst height distribution histograms in Figure 5c, collectively indicating the adsorption of proteins onto the NPs. The mean fluorescence intensity (MFI)values unveiled rapid PC formation, as nearly half of the corona proteins adsorbed within 1 min (Figure 5d). Protein adsorption reached approximately 90% in 30 min, a process aligning with the HC formation phase. Only a subtle increase in fluorescence was observed with the incubation time extended to 24 h, suggesting ongoing yet moderate protein association in the SC formation stage.^43^

**Figure 5:**
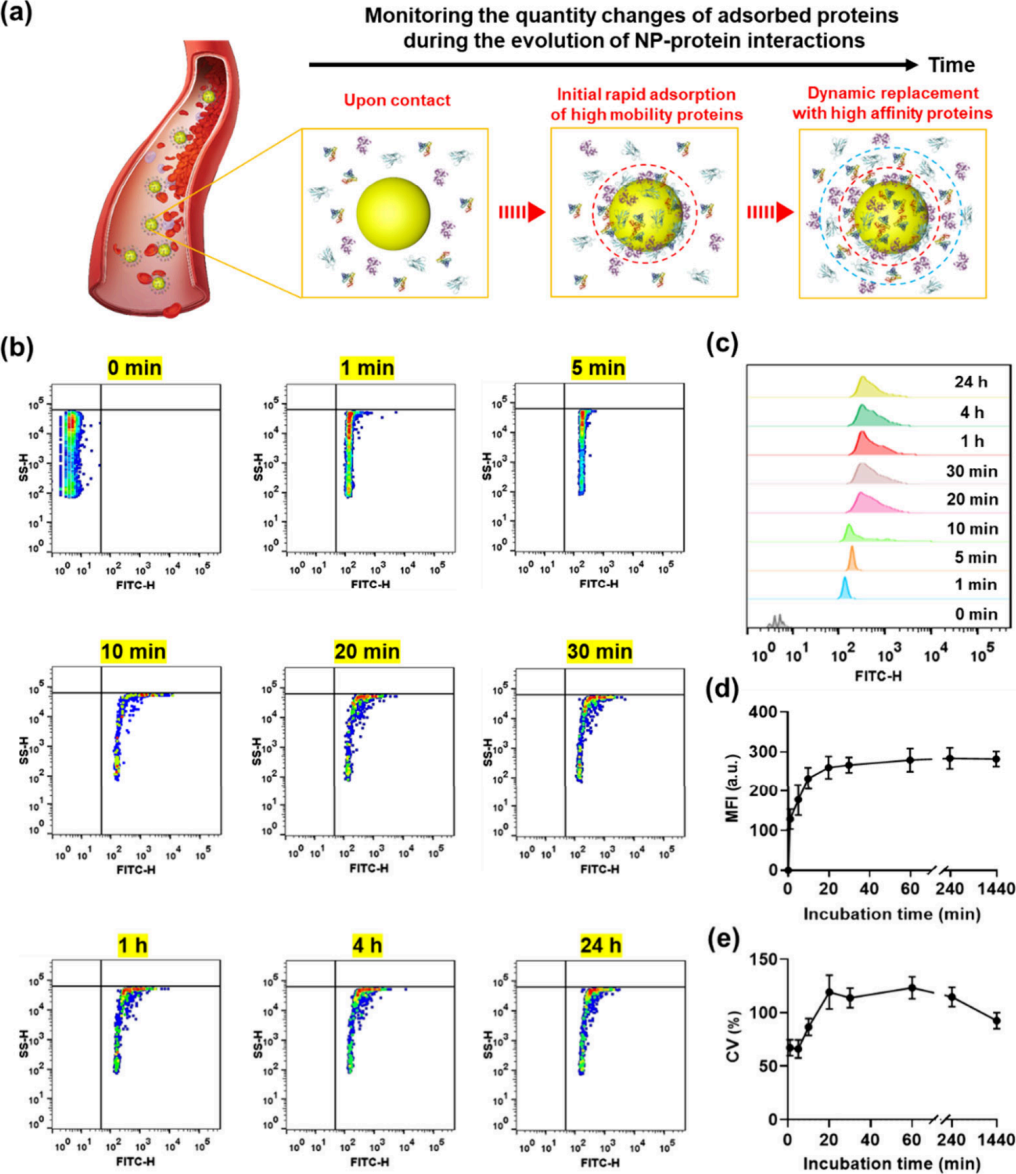
Monitoring the quantity changes of adsorbed proteins during the evolution of NP–protein interactions. (a) Schematic illustration of the evolution of protein adsorption on NPs in human serum. HC is highlighted in a red circle, while SC is differentiated by a blue circle. (b) Bivariate dot-plots of SS burst height versus FL burst height for NP–PC complexes formed over a 0 to 24 h period. (c) Histogram of FL burst area for NP–PC complexes at different times. (d) Mean fluorescence intensity of NP–PC complexes at various time intervals. (e) Coefficient of variation (CV) of the fluorescence of NP–protein complexes at different time points during serum incubation.

Figure 5c,e illustrates the evolution of heterogeneity as a function of incubation time. In Figure 5c, the MFI distribution of NP–PC complexes shows a dramatic increase after 10 min of incubation, indicating heightened heterogeneity of the NP–protein complexes. This increase in heterogeneity was further demonstrated by the CV values shown in Figure 5e. The larger CV values observed in serum protein adsorption, compared with those in single and dual protein systems (Figures 3i and 4f), indicate more complex and varied nano–bio interactions in blood serum, which may lead to distinctive *in vivo* fates among individual NPs due to their diverse protein coatings.

In summary, we developed an *in situ* approach for quantitative analysis of nanoparticle–protein interactions at the individual nanoparticle level. By utilizing scattered light for nanoparticle size evolution and fluorescence signals for quantifying protein adsorption, we identified a correlation between protein concentration and nanoparticle aggregation. Notably, low and high concentrations induced aggregation, while intermediate concentrations maintained colloidal stability through electrostatic repulsion from negatively charged proteins. Furthermore, quantifying protein adsorption unveiled a protein monolayer comprising ∼600 HSA molecules per particle. This method allowed *in situ* quantitative monitoring of nanoparticle aggregation status and protein adsorption kinetics in blood serum, therefore promising a pre-evaluation of the stability and performance of nanomedicines before *in vivo* experiments.

## Supporting Information Available

Experimental materials and methods, supplementary results, flow chart for quantification of protein adsorption on individual NPs based on NanoFCM and fluorescence measurements, calibration curves of FI against protein concentrations, protein adsorption concentrations and calculated number of HSAs adsorbed, size distribution of nanoparticles by NanoFCM, validation of effective labeling of HSA with FITC by TLC and CD spectra of HSA, SDS-PAGE gel image, TEM images of protein corona surrounding NPs, size of NP–protein complexes, optimization strategies for mitigating background fluorescence signals, *in situ* detection of protein adsorption on NPs by NanoFCM, adsorption mode of HSA on the surface of NP, and detection of protein adsorption on SiO_2_ NPs measured using NanoFCM (PDF)
