# Supplementary material for: In Situ Measurement of Nanoparticle–Blood Protein Adsorption and Its Heterogeneity with Single-Nanoparticle Resolution via Dual Fluorescence Quantification
Source: Nano Lett. 2024 Jul 22;24(30):9202–11. doi: 10.1021/acs.nanolett.4c01469 (PMC11299225; doi:10.1021/acs.nanolett.4c01469)
Supplement: Supplementary file 1 — nl4c01469_si_001.pdf [file nl4c01469_si_001.pdf]

Supporting Information

In Situ Measurement of Nanoparticle−Blood Protein Adsorption and Its Heterogeneity with Single−Nanoparticle Resolution via Dual Fluorescence Quantification

Yuanyuan Niu,[ab] Yingjie Yu,[a] Xinyang Shi,[a] Fangqin Fu,[a] Hai Yang,[c] Qiang Mu,[d] Daniel Crespy,[e] Katharina Landfester,[f]* Shuai Jiang[ab]*

[a] Key Laboratory of Marine Drugs, Chinese Ministry of Education, School of Medicine and Pharmacy, Ocean University of China, Qingdao 266003, China.

[b] Laboratory for Marine Drugs and Bioproducts, Qingdao Marine Science and Technology Center, Qingdao, 266237, China.

[c] Department of Pharmacy, Qingdao Central Hospital, University of Health and Rehabilitation Sciences, Qingdao 266042, China.

[d] The First Department of Breast Surgery, Qingdao Central Hospital, University of Health and Rehabilitation Sciences (Qingdao Central Medical Group), Qingdao 266042, China.

[e] Department of Materials Science and Engineering, School of Molecular Science and Engineering, Vidyasirimedhi Institute of Science and Technology (VISTEC), Rayong 21210, Thailand.

[f] Max Planck Institute for Polymer Research, Ackermannweg 10, 55128 Mainz, Germany.

Table of Contents

1. Experimental Section 3
1.1 Materials 3
1.2 Preparation of Buffer Solutions 4
1.3 Synthesis and Characterization of Polystyrene NPs 4
1.4 Protein Labelling 4
1.5 Preparation of Protein Corona 5
1.6 Characterization of Protein Corona 6
1.7 Quantification of Protein Corona by BCA Assay 6
1.8 Protein Corona Identification by Sodium Dodecyl Sulfate-Polyacrylamide Gel Electrophoresis (SDS-PAGE) 7
1.9 Protein Corona Analysis by NanoFCM 7
1.10 Quantification of Protein Adsorption on Individual NPs 8
2. Results 10

1. Experimental Section

1.1 Materials

Styrene (Macklin Biochemical Co., Ltd, 99%) was purified using an aluminum oxide basic column (Sinopharm Chemical Reagent Co., Ltd.). All the other chemicals and reagents were used as received without further treatment. 2-Aminoethyl methacrylate hydrochloride (AEMH, 90%), hexadecane (HD, 98%), 2,2'-azobis(2-methylbutyronitrile) (V59, 98%), sodium dodecyl sulfate (SDS, 92.5-100.5%), cetyltrimethylammonium chloride (CTAC, 97%), fluorescein 5(6)-isothiocyanate (FITC, 95%), sulfo-cyanine5 (Cy5, 99.66%), potassium chloride standard solution (KCl, 0.01 M), dimethyl sulfoxide (DMSO, >99%), dichloromethane (CH_2Cl_2, 99.5%), and methanol (CH_3OH, 99.9%) were purchased from Aladdin Biochemical Technology Co., Ltd. Sodium chloride (NaCl), sodium carbonate (Na_2CO_3), and sodium hydrogen carbonate ($NaHCO_3$) were obtained from Sinopharm Chemical Reagent Co., Ltd. Potassium chloride (KCl, 99.5%), sodium phosphate dibasic (Na_2HPO_4, 99%), potassium phosphate monobasic (KH_2PO_4, 99.5%), and dialysis cassettes (MWCO: 1.4 kDa) were purchased from Macklin Biochemical Co., Ltd. The total protein assay kit (BCA, 96T) was purchased from Nanjing Jiancheng Bioengineering Institute. SDS-PAGE precast gel, SDS-PAGE rapid electrophoresis buffer for Hepesv-Tris system, SDS-PAGE sample loading buffer (5X), and the Coomassie blue superfast staining solution were purchased from Yeasen Biotechnology Co., Ltd. Human serum albumin (HSA, 96%-99%) and transferrin (Tf, 98%) were obtained from Solarbio Science & Technology Co., Ltd. Whole blood was taken from seven healthy volunteers after physical examination and after obtaining informed consent in accordance with the Declaration of Helsinki. The study was approved by the ethic committee at Ocean University of China (Record number: OUC-HM-2022-001). Human serum was collected from all donations after clotting overnight according to a standard procedure and was pooled into one batch and stored at -80 °C. The final protein concentration was roughly 66 mg mL^{-1}, as determined by BCA assay. Milli-Q water was used in all experiments.

1.2 Preparation of Buffer Solutions

For the preparation of phosphate-buffered saline (PBS, pH = 7.4), 7.90 g of NaCl, 0.20 g of KCl, 1.44 g of Na_2HPO_4, and 0.24 g of KH_2PO_4 were dissolved in 1000 mL of Milli-Q water. The carbonate buffer saline (CBS, pH 9.6) was prepared by dissolving 9.28 g of Na_2CO_3 and 1.05 g of $NaHCO_3$ in 1000 mL of Milli-Q water.

1.3 Synthesis and Characterization of Polystyrene NPs

Synthesis of the NPs. The polystyrene NPs were prepared by miniemulsion polymerization.[1] Briefly, 252.3 mg of HD and 100.6 mg of V59 were dissolved in 5.89 g of styrene as the oil phase, while 120.0 mg of AEMH and 125.0 mg of CTAC were added to 24.0 mL of milli-Q water to form the aqueous phase. Subsequently, the aqueous phase was poured into the oil phase under stirring at 1000 rpm. The mixture was further stirred at 1000 rpm for 1 h for pre-emulsification. Next, the pre-emulsified emulsion was ultrasonicated for 3 min at 60% amplitude (Scientz 650 W sonifier with a 1/2″ tip) in an ice bath and then stirred overnight at 72 °C for polymerization. Finally, the NP dispersions were purified by dialysis against distilled water for 36 h. Silica nanoparticles (SiO_2) were prepared according to the literature.[2]

Characterization of the NPs. The hydrodynamic diameter (D_h) and zeta (ζ) potential of NPs were measured by Malvern Zetasizer (Nano-ZS ZEN3600, Malvern Instruments Co., Ltd., UK). Morphology of NPs was characterized by using transmission electron microscopy (TEM, JEM-2100EX, JEOL Ltd., Japan) and scanning electron microscopy (SEM, JSM-6360LV, JEOL Ltd., Japan).

1.4 Protein Labelling

Protein Labelling with FITC. FITC was dissolved in DMSO at a concentration of 5 mg mL^{-1} and frozen at -20 °C for long-term storage. Firstly, for HSA labelling, 10 mg of HSA powder was dissolved in 500 μL of CBS buffer. Afterward, 26 μL or 52 μL of FITC stock solution was slowly added to the HSA solution and allowed to react at room temperature for 5 h on a shaking stage. The reaction solution was further dialyzed to remove unbound FITC by dialysis against a mixture solution of DMSO and PBS (DMSO:PBS = 1:15, v/v) at 4 °C using a dialysis cassette with a MWCO of

1.4 kDa. The dialysis medium was refreshed every 12 h during the initial 36 h, followed by a subsequent 36 h period during which only PBS buffer was used. To monitor the progress of dialysis, we measured the fluorescence intensity of each dialysis solution using a microplate reader (Tecan Spark, Tecan, Switzerland). The dialysis process was continued until the fluorescence intensity of the dialysis medium reached that of PBS. In addition, thin-layer chromatography (TLC) was used to verify the successful FITC labelling by using 10 mL of CH_2Cl_2/CH_3OH (9:1 v/v) as the developing solvent. Finally, HSA-FITC was lyophilized for storage.

Fluorescently labelling of serum was performed following the protocol in literature.[3] 200 μL of original human serum was dissolved in 800 μL of CBS buffer. Subsequently, 26 μL of FITC stock solution (5 mg mL^{-1} in DMSO) was added to the serum solution and allowed to react at room temperature for 5 h on a shaking stage. The reaction mixture was then dialyzed to remove unbound FITC by dialysis against PBS at 4 °C using a dialysis cassette with a MWCO of 1.4 kDa. The dialysis process was continued until the fluorescence intensity of dialysis medium reached that of PBS.

Determining the Degree of Fluorescence Labelling. The labelling ratio (molar ratio) of FITC to proteins was determined based on independent measurements of fluorescence intensity of FITC and protein concentrations in the purified system. The FITC concentration was determined from absorbance at 495 nm on the basis of its reported extinction coefficient (ε_{495} = 76,000 M^{-1} cm^{-1}). The protein concentration was determined by using a BCA kit. Finally, we obtained HSA-FITC with labelling ratios of 0.3 and 0.6 for subsequent experiments.

1.5 Preparation of Protein Corona

The NPs were incubated in PBS solutions containing varied concentrations of HSA-FITC (0.5 mg mL^{-1}, 2 mg mL^{-1}, 5 mg mL^{-1}, and 50 mg mL^{-1}), 50 mg mL^{-1} of HSA being close to its concentration in human blood.[4] The incubation was performed at 37 ^{o}C for 1 h with a NP concentration at 1 mg mL^{-1}. The same method was also used for the incubation of NPs with HS.

For *in situ* characterization of the size of NP-protein corona complexes and quantification of protein adsorption, the incubated NP-protein solutions were directly subjected to the measurement. In order to reduce the background fluorescence signal, we used three purification methods: (1) Reduction of FITC:HSA feeding ratio from

0.6 to 0.3; (2) Dialysis for 72 h to remove unbound protein molecules; (3) Mild centrifugation (1000 g, at 4 °C, 10 min for 3 times) to remove partially the unbound proteins while preserving the protein corona composition to a maximum extent. In comparison, the hard corona was obtained by high-speed centrifugation (20000 g, 4 °C, 1 h for 3 times), in which both unbound and loosely bounded proteins were removed. The pellets were resuspended in PBS for further analysis. For *in situ* monitoring the quantity changes of adsorbed proteins during the evolution of NP-protein interactions, the incubated NP-protein solutions were directly subjected to the NanoFCM measurement.

1.6 Characterization of Protein Corona

D_h and ζ-potential of NP-protein complexes were characterized by dynamic light scattering (DLS) with a HENE laser (633 nm, 4.0 mW output power) as light source. D_h of NP-protein complexes was measured in PBS at a NP concentration of 10 μg mL^{-1}. ζ-potential of NP-protein complexes was measured in 0.001 M potassium chloride solution at a NP concentration of 10 μg mL^{-1}. Morphology of the NP-protein complexes was characterized by TEM (JEM-2100EX, JEOL Ltd., Japan). The samples (2 mg mL^{-1}) were deposited on a copper grid coated with carbon layer and then negatively stained with a 1% phosphotungstic acid solution for 3 min.

1.7 Quantification of Protein Corona by Using BCA Assay

Protein adsorption on NPs was quantified using a Pierce BCA protein assay. Briefly, the bounded proteins were firstly desorbed from NPs by incubating the NP-protein complexes with 100 μL SDS-Tris buffer (2% SDS, 62.5 mM Tris-HCl) at 95 °C for 5 min. The detached proteins were separated from NPs by centrifugation (14000 rpm for 1 h at 4 °C). The supernatant was recovered for BCA assay according to the manufacturer's instructions. The number of HSA molecules adsorbed on NPs has been theoretically estimated by considering the protein as rigid sphere with a diameter (d) of 7.5 nm and a cross-sectional area (σ) of 44.16 nm^2. The maximum adsorbed number of HSA molecules per NP, corresponding to a close-packed arrangement of the protein on the NP surface,[5] was 1600 for NPs with a diameter of 150 nm.

1.8 Protein Corona Identification by Sodium Dodecyl Sulfate-Polyacrylamide Gel Electrophoresis (SDS-PAGE)

The presence of proteins on NP surface was verified by SDS-PAGE electrophoresis. The above-mentioned recovered protein solutions from protein desorption were mixed with protein loading buffer and then boiled at 100 °C for 10 min. After that, the sample was loaded in 4-20% SDS-PAGE gel. Gel electrophoresis was performed at 150 V for 45 min until the proteins with the lowest molecular weight near the end of the gel. The protein patterns were visualized after Coomassie blue staining.

1.9 Protein Corona Analysis by NanoFCM

The side scattering and fluorescence intensity of each NP-protein complexes sample were measured *via* Flow NanoAnalyzer (NanoFCM INC) to characterize *in situ* the protein corona formation at single nanoparticle level. With distilled water as the sheath fluid and using a 488 nm laser diode excitation wavelength, the side scattered light and fluorescence signals were collected in separate channels using avalanche photodiode detectors (APD). Background measurement in PBS buffer was performed before each measurement series. The threshold levels for both the peak height (a digital discriminator level set to 3 times the standard deviation of the background) and the peak width (0.2 ms) were used as the criteria for burst (or peak) identification. For each burst that satisfied the criteria, the integrated number of photons (background subtracted) was stored as the burst area for the histogram or dot-plot construction. The results were presented as a mean of the total fluorescence intensity of the FITC-labelled proteins. Unless stated otherwise, each distribution histogram or dot-plot was derived from data collected over 1 min. All the experiment results were processed by NF Profession v2.0, FCS.3.0, and analyzed with GraphPad Prism 8.0 software.

1.10 Quantification of Protein Adsorption on Individual NPs

The procedure for determining the number of proteins on individual NPs is explained in the following flow chart (**Scheme S1**).

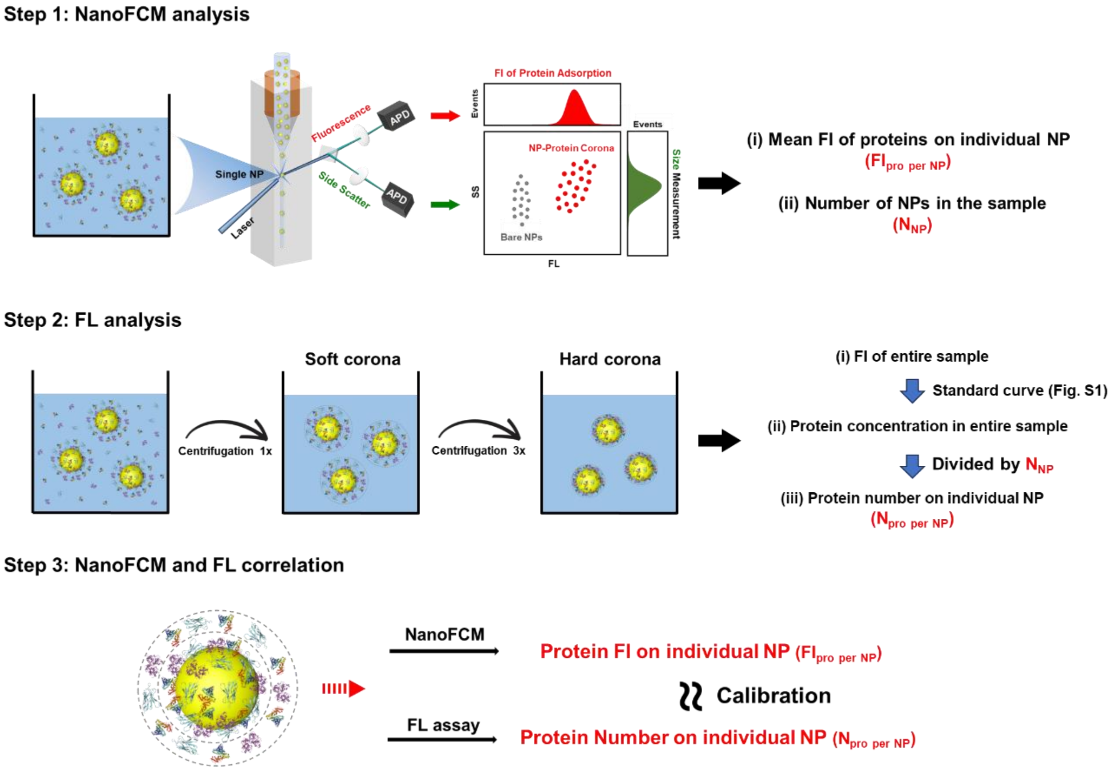

Scheme S1. Flow chart for quantification of protein adsorption on individual NPs based on NanoFCM and fluorescence measurements.

Step 1 – NanoFCM analysis: Fluorescence intensity (FI) of fluorescent proteins adsorbed on individual NPs (i) and NP concentration (ii) were measured using NanoFCM.

Step 2 – Fluorescence (FL) analysis: To convert the FI data obtained from NanoFCM to the number of proteins, the same samples (as step 1) were analyzed using microplate reader after a centrifuge step to remove unbound proteins. The NP-protein incubation mixture was centrifuged once to obtain NP-soft corona complexes, or washed three times by centrifugation for hard corona analysis.

(i) Overall FI of proteins in entire sample was detected by using microplate reader;

(ii) The FI data was converted to protein concentration by using a calibration curve of FI against protein concentration (Fig. S1).

(iii) Protein amount on individual NPs was calculated according to following

equation (1):

$$C_{n[protein]} = \frac{C_{m[protein]}}{MW_{protein}} \cdot N_A \quad (1)$$

$$N_{protein\ per\ NP} = \frac{C_{n[protein]}}{N_{NP}} \quad (2)$$

where $C_{n[protein]}$ is the number concentration of protein, $C_{m[protein]}$ is the mass concentration of protein, $M_{W\ protein}$ is molecular weight of protein, N_A is the Avogadro number ($6.023 \cdot 10^{23}$), and N_{NP} is the number concentration of NPs.

Step 3 – Correlation of NanoFCM and FL analysis: Based on the results of steps 1 and 2, the FI data on individual NPs from NanoFCM is calibrated using the FL results.

2. Results

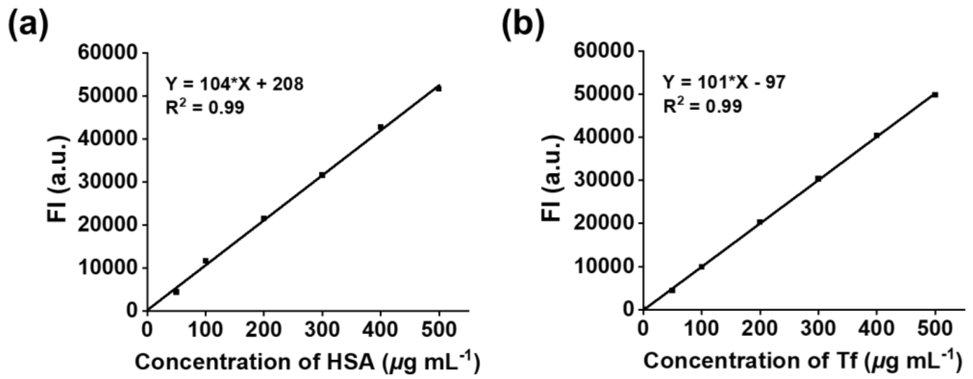

Figure S1. Calibration curves of FI against protein concentrations obtained by microplate reader: (a) HSA-FITC, (b) Tf-Cy5.

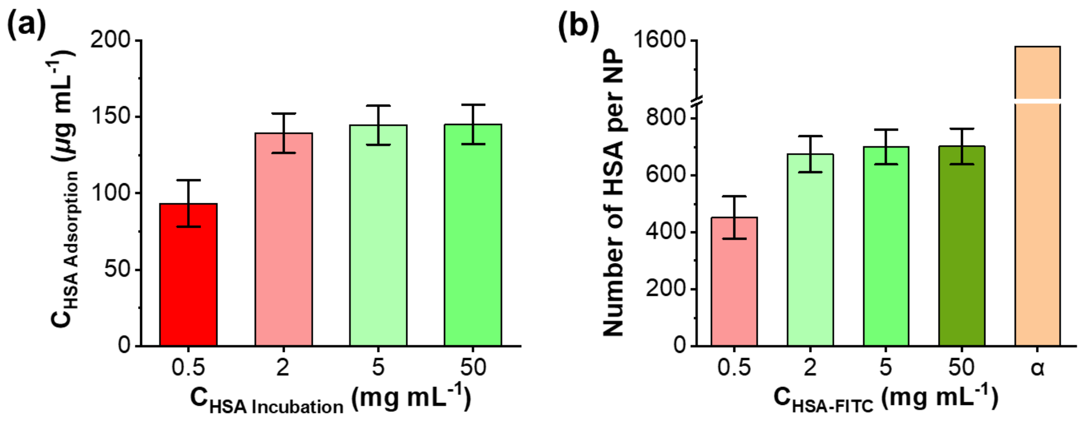

Figure S2. (a) Protein adsorption concentrations resulting from different incubation concentrations of HSA, detected by BCA assay. (b) Calculated number of HSA adsorbed per NP. The corresponding NP concentration is $1.86 \cdot 10^{12}$ particles per mL obtained from NanoFCM.

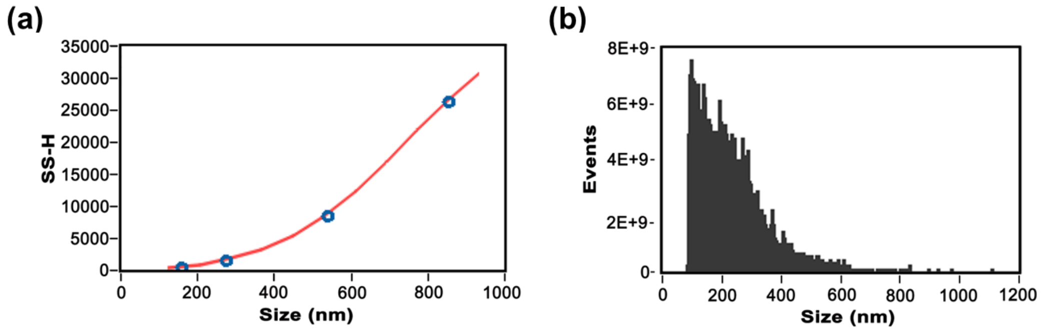

Figure S3. Size distribution of nanoparticles by NanoFCM. (a) Standard curve of intensity of side scattered light against size of standard NPs (diameters = 155 nm, 270 nm, 535 nm, or 850 nm). The curve was fitted with the equation $y = 41177/(1 + 84e^{-0.006x}) - 634$. $R^2 = 0.9999$. (b) Histogram of NP size distribution.

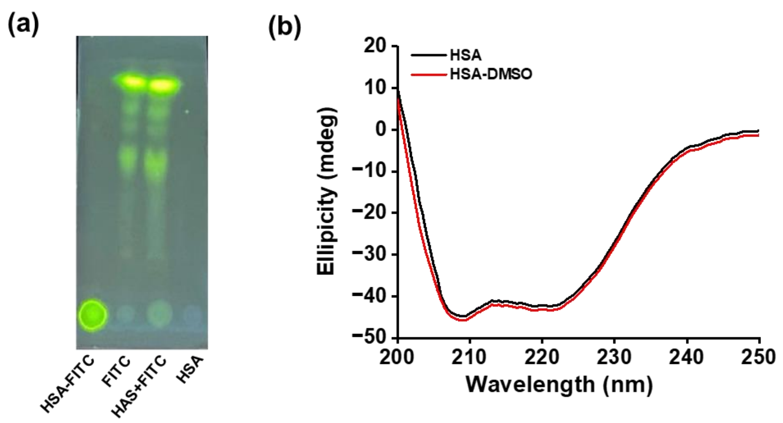

Figure S4. (a) Validation of effective labelling of HSA with FITC by TLC. The first column represents the sample of FITC and HSA reaction in CBS buffer at 37 °C for 5 h. The second and fourth columns represent the free FITC and HSA. The third column HSA+FITC represents the sample immediately subjected to TLC after mixing HSA and FITC in the PBS buffer. (b) CD spectra of HSA in PBS and PBS containing 10 vol% DMSO.

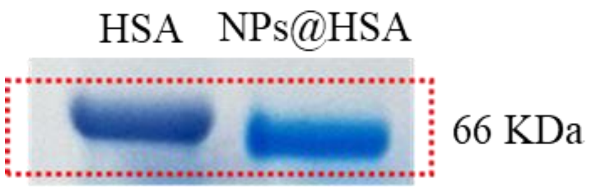

Figure S5. SDS-PAGE gel image of protein bands of pure HSA and detached HSA from NP-protein complexes.

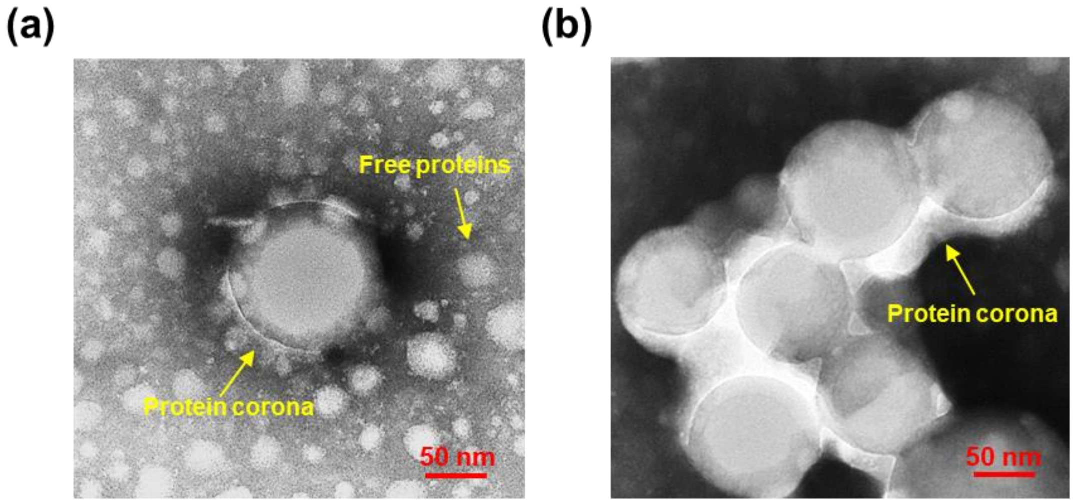

Figure S6. TEM images of protein corona surrounding NPs. Protein corona after incubating NPs with HSA at a concentration of 5 mg mL^{-1} without purification (a), and after centrifugation to remove the free proteins (b). The white dots represent aggregates of free HSA. Phosphotungstic acid was used for staining.

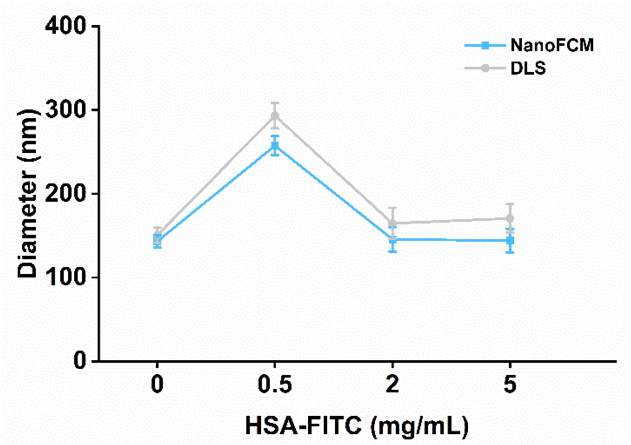

Figure S7. The size of NP-protein complexes before and after incubation with HSA-FITC at different concentrations detected by NanoFCM and DLS.

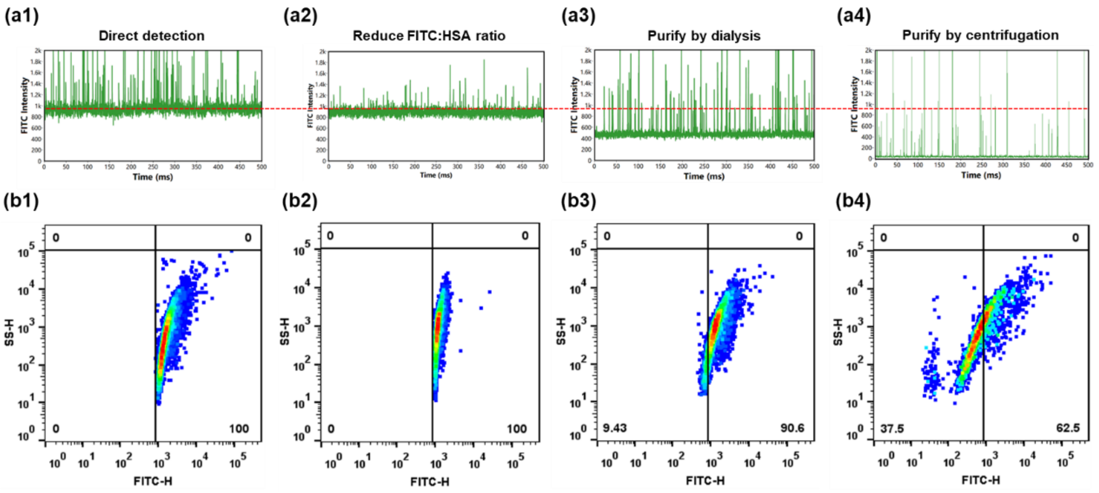

Figure S8. Optimization strategies for mitigating background fluorescence signals. (a) FL signals of NPs upon incubation with 5 mg mL^{-1} HSA-FITC. Three strategies, including (i) reducing FITC labeling ratio, (ii) purifying the NP-protein mixture through dialysis, and (iii) purifying the NP-protein mixture *via* centrifugation, were evaluated for their effectiveness in mitigating background fluorescence signals. (b) Corresponding bivariate dot-pots of SS burst height versus FL burst height.

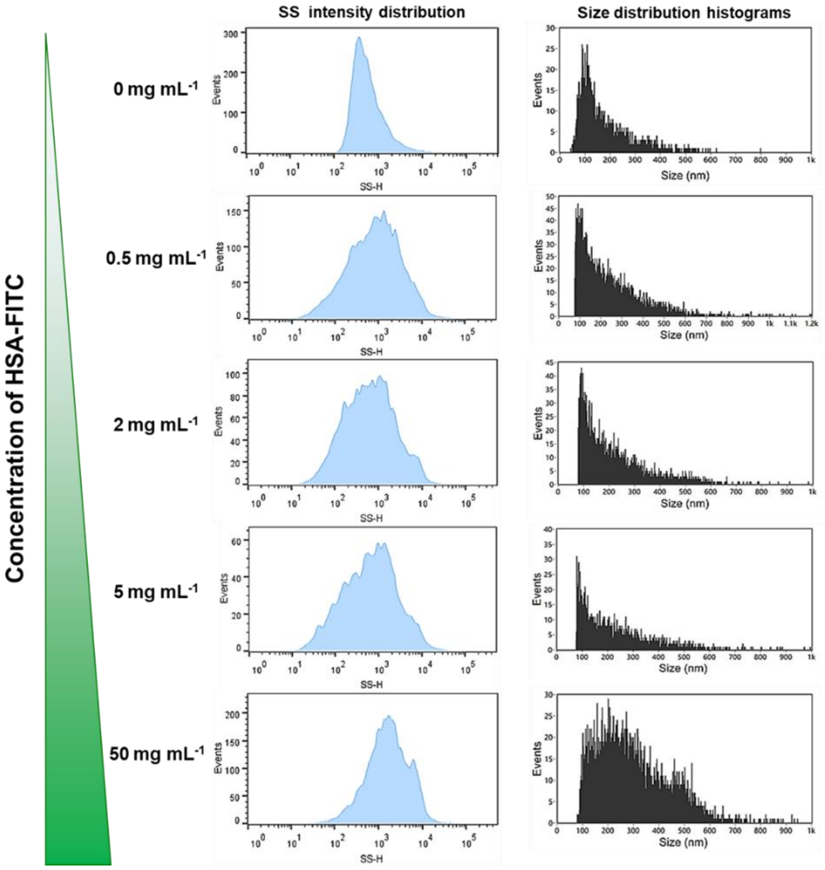

Figure S9. *In situ* detection of protein adsorption on NPs by NanoFCM. SS analysis of NPs before and after incubation with varying concentrations of HSA-FITC (0 mg mL^{-1}, 0.5 mg mL^{-1}, 2 mg mL^{-1}, 5 mg mL^{-1} or 50 mg mL^{-1}) for 1 h, including SS intensity distribution histograms, and size distribution histograms.

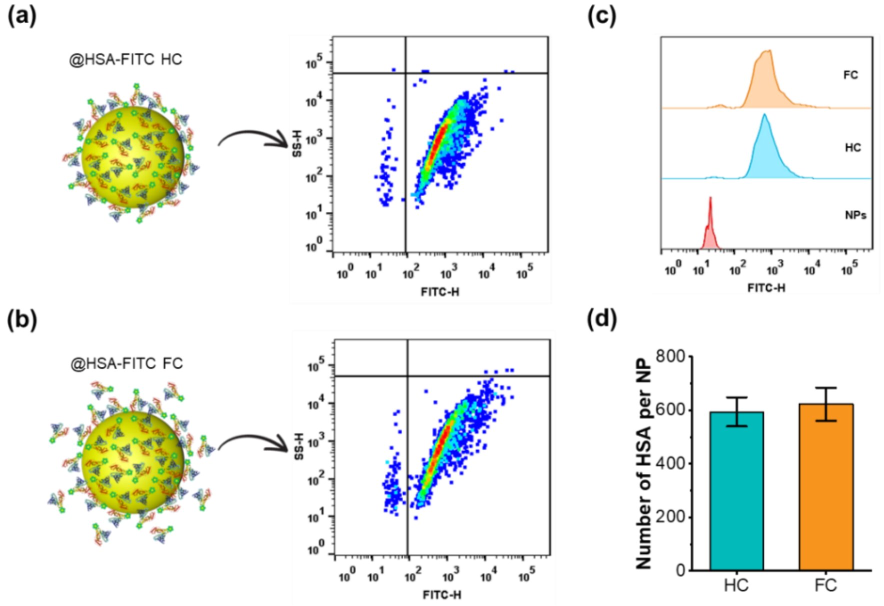

Figure S10. Adsorption mode of HSA on the surface of NP. (a) Bivariate dot-plot of SS signals versus FL signals of NP@HSA-FITC HC. (b) Bivariate dot-plot of SS signals versus FL signals of NP@HSA-FITC FC. (c) Histograms represent the FL burst height distribution of FC and HC. (d) Calculated number of HSA per NP in the HC and FC.

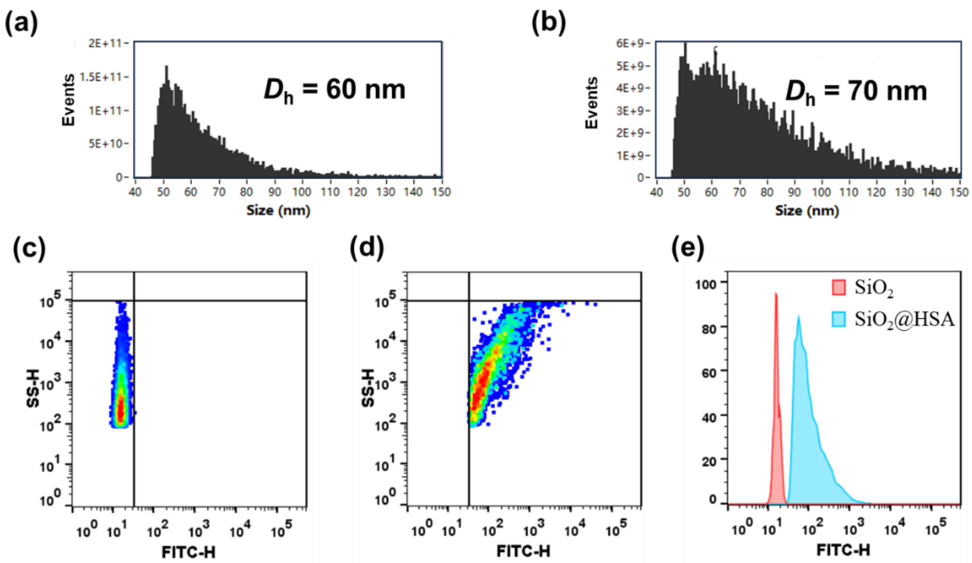

Figure S11. Detection of protein adsorption on SiO_2 NPs measured using NanoFCM. (a) Histogram of SiO_2 NP size distribution. (b) Histogram of SiO_2@HSA size distribution. (c) Bivariate dot-plots of SS signals versus FL signals of SiO_2 NPs. (d) Bivariate dot-plots of SS signals versus FL signals of SiO_2@HSA. (e) Histograms of FL intensity distribution.

References

(1) Landfester, K. Synthesis of colloidal particles in miniemulsions. *Annu. Rev. Mater. Res.* **2006,** *36* (1), 231-279.

(2) Ding, H.; Zhang, Y.; Wang, S.; Xu, J.; Xu, S.; Li, G. Fe_3O_4@SiO_2 core/shell nanoparticles: the silica coating regulations with a single core for different core sizes and shell thicknesses. *Chem. Mater.* **2012,** *24* (23), 4572-4580.

(3) Bertoli, F.; Garry, D.; Monopoli, M. P.; Salvati, A.; Dawson, K. A. The intracellular destiny of the protein corona: a study on its cellular internalization and evolution. *ACS nano* **2016,** *10* (11), 10471-10479.

(4) Kelly, P. M.; Åberg, C.; Polo, E.; O'connell, A.; Cookman, J.; Fallon, J.; Krpetić, Ž.; Dawson, K. A. Mapping protein binding sites on the biomolecular corona of nanoparticles. *Nat. Nanotechnol.* **2015,** *10* (5), 472-479.

(5) Hlady, V.; Buijs, J. Protein adsorption on solid surfaces. *Curr. Opin. Biotechnol.* **1996,** *7* (1), 72-77.
